# Genome-wide association and RNA-seq analyses reveal genes linked to salt stress in peanut (*Arachis hypogaea* L.)

**DOI:** 10.3389/fpls.2025.1699469

**Published:** 2025-11-27

**Authors:** Kunyan Zou, Yang Jae Kang, Bo-Keun Ha, Kyung Do Kim, Ki-Seung Kim, Tae-Hwan Jun

**Affiliations:** 1 Department of Machinery and Automation, Weifang University, Weifang, China; 2 Division of Bio & Medical Bigdata Department (Brain Korea 21 Four), Gyeongsang National University, Jinju, Republic of Korea; 3 Research Institute of Molecular Alchemy, Gyeongsang National University, Jinju, Republic of Korea; 4 Department of Applied Plant Science, Chonnam National University, Gwangju, Republic of Korea; 5 Department of Integrative Biological Sciences and Industry, Sejong University, Seoul, Republic of Korea; 6 Institute for Advanced Plant Breeding and Phytochemicals (IAPBP), Sejong University, Seoul, Republic of Korea; 7 New Agrochemicals Research Institute, FarmHannong, Ltd., Nonsan, Republic of Korea; 8 Department of Plant Bioscience, Pusan National University, Miryang, Republic of Korea; 9 Life and Industry Convergence Research Institute, Pusan National University, Miryang, Republic of Korea

**Keywords:** peanut, GWAS, RNA-seq, salt stress, candidate genes

## Abstract

Salt stress adversely affects seed germination, seedling growth, and development, considerably impacting peanut (*Arachis hypogaea* L.) production. However, their genetic and genomic responses to salinity remain poorly understood. To identify candidate genes associated with salt tolerance, a genome-wide association study (GWAS) of 295 peanut genotypes and RNA sequencing (RNA-seq) analysis of two contrasting accessions (tolerant and susceptible) exposed to 200 mM NaCl at the seedling stage were conducted. Leaf scorch, sodium ion concentration, proline content, and chlorophyll content were evaluated as primary indicators of salt tolerance. GWAS identified 10 single-nucleotide polymorphisms significantly associated with salt stress. Transcriptome analysis of root tissues revealed 1,734 differentially expressed genes, significantly enriched in pathways such as oxidoreductase activity, defense response, flavonoid biosynthesis, transcription factor activity, and cytochrome P450-related functions. Seventeen common candidate genes were identified through the integration of GWAS and RNA-seq results. Of these genes, seven exhibited expression levels significantly correlated with relevant salt tolerance traits. Sequence variations were detected in two of the seven genes, associated with sodium ion content and leaf scorch score, respectively. Using validated mutation data, we developed a kompetitive allele-specific polymerase chain reaction marker to assess proline levels, which enable breeders to make precise and early selections at the field level, thereby reducing both the time and cost required for developing new salt-tolerant varieties through efficient marker-assisted selection. Our integrated genomic and transcriptomic analysis identified seven high-confidence candidate genes, providing new insights and theoretical basis for cloning salt-tolerant genes. These findings advance understanding of the molecular mechanisms underlying peanut adaptation to salt stress and offer valuable genetic resources, including tolerant accessions and associated or linked genomic regions, to support breeding programs for developing salt-tolerant cultivars.

## 1 Introduction

Peanut (*Arachis hypogaea* L.), also known as groundnut, is an important oilseed and legume crop (Zhao et al., 2025). Global peanut cultivation spans approximately 30.9 million hectares with a production volume of 54.3 million metric tons, making it a crucial crop for both food security and edible oil supply (FAO STAT, 2024). Soil salinization is a growing global concern driven by land degradation, climate change, and drought (Satu et al., 2019). Excessive soil salinity imposes osmotic stress and ion toxicity (Yang and Guo, 2018a, b), which disrupt photosynthesis, reduce yield potential, and may ultimately lead to plant death (El-Akhal et al., 2013; Mickelbart et al., 2015; Munns and Tester, 2008; Qin et al., 2011; Sui et al., 2018). As a widely cultivated legume crop, peanut is frequently exposed to saline conditions in major production regions. Therefore, understanding the genetic basis of salt tolerance in peanut is critical for identifying tolerant genotype, elucidating underlying molecular mechanisms, and developing high-yielding cultivars with enhanced adaptability to saline-alkali soils, thereby supporting sustainable production and contributing to global food security (Xu et al., 2022).

Cultivated peanut is an allotetraploid (AABB), and its genetic diversity is particularly low due to polyploidization (Kim et al., 2017), which complicates conventional genetic analysis (Bertioli et al., 2019). Although the release of the genome sequences of the diploid ancestors and cultivated peanut (Chen et al., 2016b, 2019) has greatly advanced genetic and genomic research in peanut, studies specifically targeting salt tolerance remain limited. High-density SNP arrays have facilitated high-resolution genetic mapping, genomic selection, and genome-wide association studies (GWASs) in peanut (Pandey et al., 2012, 2016; Varshney et al., 2013), thereby accelerating progress in genetic and genomic researches and breeding programs. Previous GWAS in peanut have successfully identified loci for agronomic traits including yield, quality, and seed composition using high-density SNP arrays (Pandey et al., 2012; Wang et al., 2019; Zhang et al., 2021). However, to the best of our knowledge, no GWAS has been conducted in peanut to identify genetic loci or candidate genes involved in salt stress tolerance.

In contrast, recent RNA-seq studies have made significant progress in elucidating key genes involved in the salt stress response of peanut through integrated multi-omics analyses. Wang et al. (2024) identified several genes encoding LEA proteins that were significantly induced under salt stress. Subsequent functional analyses demonstrated that NF-YC family transcription factors (AhNF-Y) enhance salt tolerance by regulating stomatal aperture and osmotic adjustment–related genes (Wan et al., 2021). Furthermore, Yang et al. (2023) reported that the *AhSAMS1* gene regulates ion homeostasis, and its loss of function results in increased sodium accumulation and reduced salt tolerance. The most recent study by Mou et al. (2025) revealed that plants overexpressing AhOPR6 exhibited significantly enhanced resistance to salt stress, with the AhOPR6-OE lines showing a higher capacity to scavenge reactive oxygen species (ROS), suggesting that *AhOPR6* may serve as a promising candidate gene for improving salt tolerance in peanut through genetic transformation.

However, most transcriptome studies have been conducted without the complementary power of genetic mapping, making it difficult to distinguish core regulatory genes from secondary responses and to pinpoint causative genetic variants (Zhang et al., 2024). Moreover, major challenges associated with RNA-seq include the large volume of data required as well as the complexity of data analysis and interpretation (Deshpande et al., 2023; McDermaid et al., 2019). The complexity of the peanut genome and its incomplete annotation further complicate transcriptomic analyses compared with those in other crops (Bhat et al., 2021). Consequently, a comprehensive and integrative framework linking transcriptomic changes to genomic regions under salt stress remains lacking. Therefore, a single methodological approach is insufficient to overcome these limitations. GWAS enables the identification of genomic regions statistically associated with phenotypic variation in salt tolerance across diverse genotype, thereby pinpointing loci potentially under evolutionary selection (Li and Ritchie, 2021). Concurrently, RNA-seq provides insights into dynamic transcriptional reprogramming in response to salt stress, revealing key pathways and differentially expressed genes (Garg, 2021). By integrating these two datasets, candidate genes can be prioritized that are not only located within significant GWAS loci but also exhibit salt-responsive expression patterns (Kim and Kim, 2023). This convergence of genomic and transcriptomic evidence substantially enhances the likelihood of identifying functionally relevant genes, thereby overcoming the inherent limitations of either method when used in isolation.

Kompetitive allele-specific PCR (KASP) is a competitive allele-specific PCR method based on dual fluorescent resonance energy transfer offer such a solution (Alvarez-Fernandez et al., 2021). KASP markers offer several advantages, including high throughput, sensitivity, specificity, and low cost, developing KASP markers from significant SNPs associated with salt tolerance would enable efficient marker-assisted selection. This method has been widely utilized in SNP genotyping studies of crops such as rice (Sandhu et al., 2022), soybean (Rosso et al., 2021), wheat (Grewal et al., 2022), and peanut (Zhao et al., 2017).

In the context of salt stress tolerance in peanut, candidate genes and underlying genetic mechanisms have not been thoroughly investigated; no salt-tolerance gene has been made available for use in breeding programs. Therefore, the identification of candidate genes and genetic mechanisms associated with salt stress response is essential to advance future peanut breeding efforts. In the present study, both GWAS and RNA-seq were utilized to identify candidate genes for salt tolerance and select salt-tolerant genotypes at the seedling stage. Additionally, KASP markers based on significant SNPs were developed to evaluate their effectiveness in distinguishing salt-tolerant peanut accessions. This study is expected to provide valuable molecular information for researchers investigating salt tolerance in peanut and to support the development of improved varieties by supplying breeders with salt-tolerant accessions.

## 2 Materials and methods

### 2.1 Plant materials and phenotypic analysis

In total, 295 peanut accessions were used for GWAS in this study (**Supplementary Table S1**). The accessions were planted in the greenhouse at Pusan National University, Miryang, South Korea, in August 2021. Five biological replicates were established for each accession, and three similar plants were randomly selected per accession for phenotypic data collection. Each plant was grown in a 5cm × 5cm seedling dish under a photoperiod of 14h light/10h dark at a constant temperature of 26°C. At the 1-month seedling stage, plants were treated with half-strength Hoagland solution containing 200 mM NaCl (treated group) or with solution lacking NaCl (control group; 0 mM NaCl) to induce salt stress. After 10 days of treatment, leaf scorch score (LSS), and leaf chlorophyll content were measured. Sodium ion content, and proline content were measured after plant material had been dried at 55°C for 72h.

**Figure 1:**
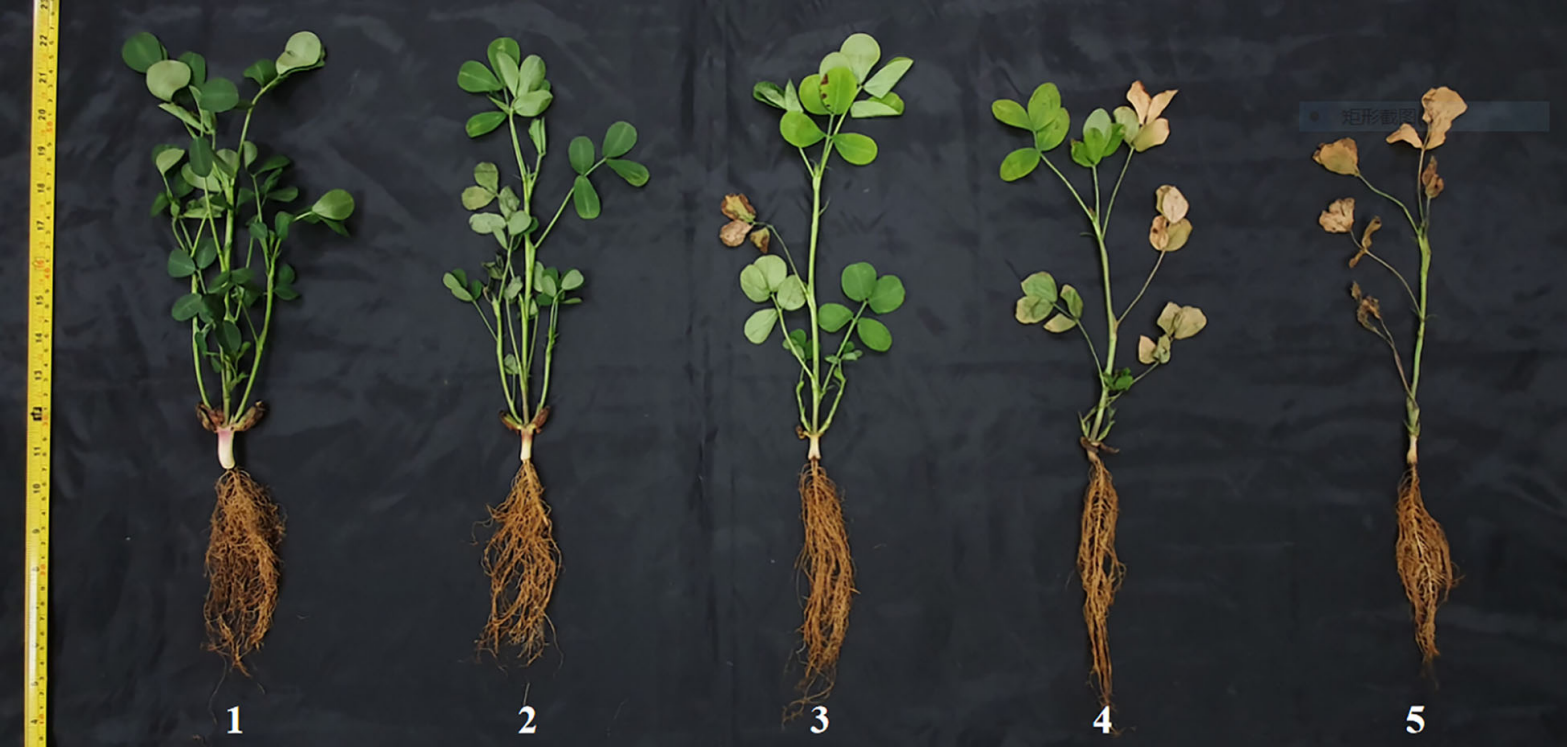
Leaf scorch score (LSS) was scored using a 1–5 scale. From left to right are 1 = no apparent chlorosis; 2 = slight (25% of the leaves showed chlorosis); 3 = moderate (50% of the leaves showed chlorosis and some necrosis); 4 = severe chlorosis (75% of the leaves showed chlorosis and severe necrosis); and 5 = dead (leaves showed severe necrosis and were withered).

LSS was assessed using a 1–5 scale (**Figure 1**): 1 = no apparent chlorosis; 2 = slight chlorosis (25% of leaves affected); 3 = moderate chlorosis (50% of leaves affected with some necrosis); 4 = severe chlorosis (75% of leaves affected with pronounced necrosis); and 5 = dead (leaves exhibited severe necrosis and complete withering) (Do et al., 2019). Leaf chlorophyll content was measured three times on the first fully opened trifoliate leaf of each peanut plant using a SPAD-502 Plus chlorophyll meter (Markwell et al., 1995; Samdur et al., 2000), and the mean value obtained from three plants per accession was used for subsequent analysis. Sodium ion content was measured in three individuals per accession with comparable growth. Dried tissue was ground using a CMT Vibrating Sample Mill (Kim et al., 2021). A 0.1-g sample of ground tissue was mixed with 1 mL of distilled water and shaken for 1min. A 0.5-mL aliquot of the mixture was transferred to a LAQUAtwin Na^+^ sensor using a pipetting gun. Sodium concentration was recorded after the value stabilized, and each sample was measured in duplicate. Proline content was determined by extracting tissue with sulfosalicylic acid. Pigments were extracted using toluene after heating with acid ninhydrin. Absorbance was measured at 520 nm using colorimetry, and proline concentration was calculated using a standard curve (Du et al., 2018) with an Epoch™ Microplate Spectrophotometer.

Based on preliminary phenotypic evaluation of the 295 peanut accessions, GWP331 (T, salt-tolerant) and GWP161 (S, salt-susceptible) were selected for RNA-seq analysis and were planted in the greenhouse at Pusan National University, Miryang, South Korea, in September 2020. For transcriptome profiling, root tissues from three biological replicates of control and NaCl-treated plants were collected at 0, 12, and 24h post-treatment, flash-frozen in liquid nitrogen, and stored at –80°C for RNA extraction.

### 2.2 Genome-wide association study

The high-density SNP array Axiom_Arachis, containing 58,000 SNPs, was used to generate genotypic data (Zou et al., 2020). The reference genome arahy.Tifrunner.gnm1.KYV3 served as the reference for array design. GWAS was conducted via the GAPIT (version 3) (Wang and Zhang, 2021) package in R software, the VanRaden method was used to calculate the kinship matrix, using the enriched compressed mixed linear model (ECMLM) to assess associations between SNPs and phenotypic traits. The Bonferroni-corrected p-value (1/total number of SNPs) was utilized to determine statistical significance. To evaluate genotype frequencies at significant loci, 10 peanut genotypes with the highest salt tolerance and 10 peanut genotypes with the highest salt susceptibility were selected. Associations between selected SNPs and phenotypic traits were explored using t-tests, and the results were visualized using Origin software. Linkage disequilibrium (LD) analysis was performed on SNP pairs with a minor allele frequency greater than 0.01. Haploview v4.2 (Barrett et al., 2005) was used to visualize LD blocks. LD strength was expressed as *r*^2^, the square of the correlation coefficient between two indicator variables. Candidate genes located within 500 kb upstream or downstream of peak SNPs, based on LD block structure, were selected using the National Center for Biotechnology Information (NCBI) reference genome.

### 2.3 RNA-seq and transcription analysis

RNA was extracted from fresh root tissues of T and S after treatment with 200 mM NaCl for 0, 12, and 24h. RNA extraction was carried out using the Qiagen RNeasy Plant Mini Kit, and genomic DNA was removed using Qiagen RNase-Free DNase. RNA concentration and purity were assessed with a NanoDrop ND-1000 spectrophotometer, and RNA integrity was verified by 1% agarose gel electrophoresis. These samples were used to construct sequencing libraries, and genome sequencing was performed using the Illumina NovaSeq 6000 platform by DNA Link Co., Ltd. (Seoul, South Korea). Raw reads were initially assessed using FastQC v0.12 for quality control. Trimmomatic v0.36 (Bolger et al., 2014) was then used to remove adapter sequences, low-quality bases, and short reads. Clean reads were mapped to the *A. hypogaea* reference coding sequence genome using Kallisto v0.46 with the Spearman rank correlation method (Bray et al., 2016). The Kallisto index was constructed using the reference transcriptome.

Transcript-level count values were imported and summarized at the gene level using the Tximport package (v1.28) (Soneson et al., 2016) in R software. Differential expression analysis was performed using the DESeq2 package (v1.40) (Love et al., 2014) in R software to identify differentially expressed genes (DEGs) across transcriptome groups. DEGs were selected based on a false discovery rate ≤ 0.05 and absolute log_2_ fold change (|log_2_FC|) > 2, with upregulation defined as log_2_FC > 2 and downregulation as log_2_FC < –2 (Anders and Huber, 2010). Candidate genes were further filtered using a –log_10_ p-value threshold > 15. K-means clustering was implemented using the row_km function in the ComplexHeatmap package (Gu et al., 2016) (in R software) to group and visualize expression patterns; data representation was conducted through ClusterGVis. Gene Ontology (GO) enrichment analysis was performed using reference GO annotations (Ashburner et al., 2000). The significance threshold was set at p ≤ 0.05; GO terms were categorized into biological process, cellular component, and molecular function domains. Functional annotation was conducted using BLASTP with amino acid sequences aligned against the Kyoto Encyclopedia of Genes and Genomes (KEGG) database (Ogata et al., 1999), considering best hits at p ≤ 0.05. Venn diagrams were used to illustrate and compare expressed sequences shared across different samples. A gene co-expression network was constructed via Cytoscape v3.4 (Shannon et al., 2003).

### 2.4 Quantitative Reverse Transcription PCR (qRT-PCR)

cDNA was synthesized using the SuperScript™ III First-Strand Synthesis System. qRT-PCR was performed using PowerUp SYBR Green Master Mix on the Applied Biosystems QuantStudio 1 Real-Time PCR System. Each reaction was conducted in triplicate. Primer pairs for the target genes were designed using the NCBI Primer-BLAST tool. Transcript levels were normalized to the expression of the actin gene (forward primer: 5′-TACCAGATGGACAGGTTATCACAAT-3′; reverse primer: 5′-TGGAACCACCACTCAAGACAAT-3′). Each 20-μL amplification reaction contained 10 μL PowerUp SYBR Green Master Mix, 1 μL cDNA, 10 pmol/μL of each specific primer, and 7 μL nuclease-free water. PCR cycling conditions were as follows: initial denaturation at 95°C for 10 min, followed by 40 cycles of 95°C for 15 s, 60°C for 15 s, and 72°C for 1 min. Melting curve analysis was performed at 95°C for 15 s, 60°C for 1 min, and 95°C for 15 s to confirm amplification specificity. Relative gene expression levels were calculated using the 2^−ΔΔCt^ method; actin served as the internal reference control. Statistical comparisons were performed using an unpaired two-tailed Student’s t-test at a significance level of p < 0.05.

### 2.5 Development of KASP markers

KASP assay design was performed by a service provider in accordance with guidelines from Biosearch Technologies, then aligned with corresponding SNPs from the Axiom_Arachis array. The KASP assay was used to differentiate 30 genotypes with the highest and lowest sodium ion content among the 295 accessions. For SNP AX-147250101, the allele-specific primers included FAM (5′-TGTGTCATAAGGAGCCATCAAGAGTT-3′) and HEX (5′-TGTCATAAGGAGCCATCAAGAGTC-3′) tails, with the corresponding reverse primer: 5′-GTCGCCCTGATATGCAAGCATAGTT-3′. Each 10-μL KASP reaction contained 5 μL KASP master mix (2×), 50 ng genomic DNA template, and 0.14 μM of allele-specific primer mix. PCR amplification was carried out under the following conditions: initial denaturation at 94°C for 15 min; 10 touchdown cycles of 94°C for 20 s and annealing starting at 61°C (decreasing by 0.6°C per cycle) for 60 s; followed by 26 additional cycles of 94°C for 20 s and 55°C for 60 s. After amplification, plates were subjected to fluorescence evaluation; allele calls were generated using QuantStudio™ Design & Analysis v1.5.1.

### 2.6 Sequencing of candidate genes

Candidate genes were sequenced from the T and S. PCR amplification was performed using the GoTaq^®^ G2 Green Master Mix for Sanger sequencing. Each 50-μL reaction mixture contained 25 μL GoTaq^®^ G2 Green Master Mix, 0.1 μM of each primer, 30 ng DNA template, and nuclease-free water to reach 50 μL total volume. PCR conditions comprised initial denaturation at 95°C for 5 min, followed by 38 cycles of 95°C for 30 s, 60°C for 30 s, and 72°C for 2 min. Amplified products were purified using the FavorPrep™ GEL/PCR Purification Mini Kit, in accordance with the manufacturer’s instructions. Sequencing of PCR products using external forward or reverse primers was performed on an ABI 3730XL Genetic Analyzer. The resulting sequences were analyzed using SnapGene v6.2.0.

## 3 Results

### 3.1 Physiological responses to salt stress in peanut genotypes

**Figure 2:**
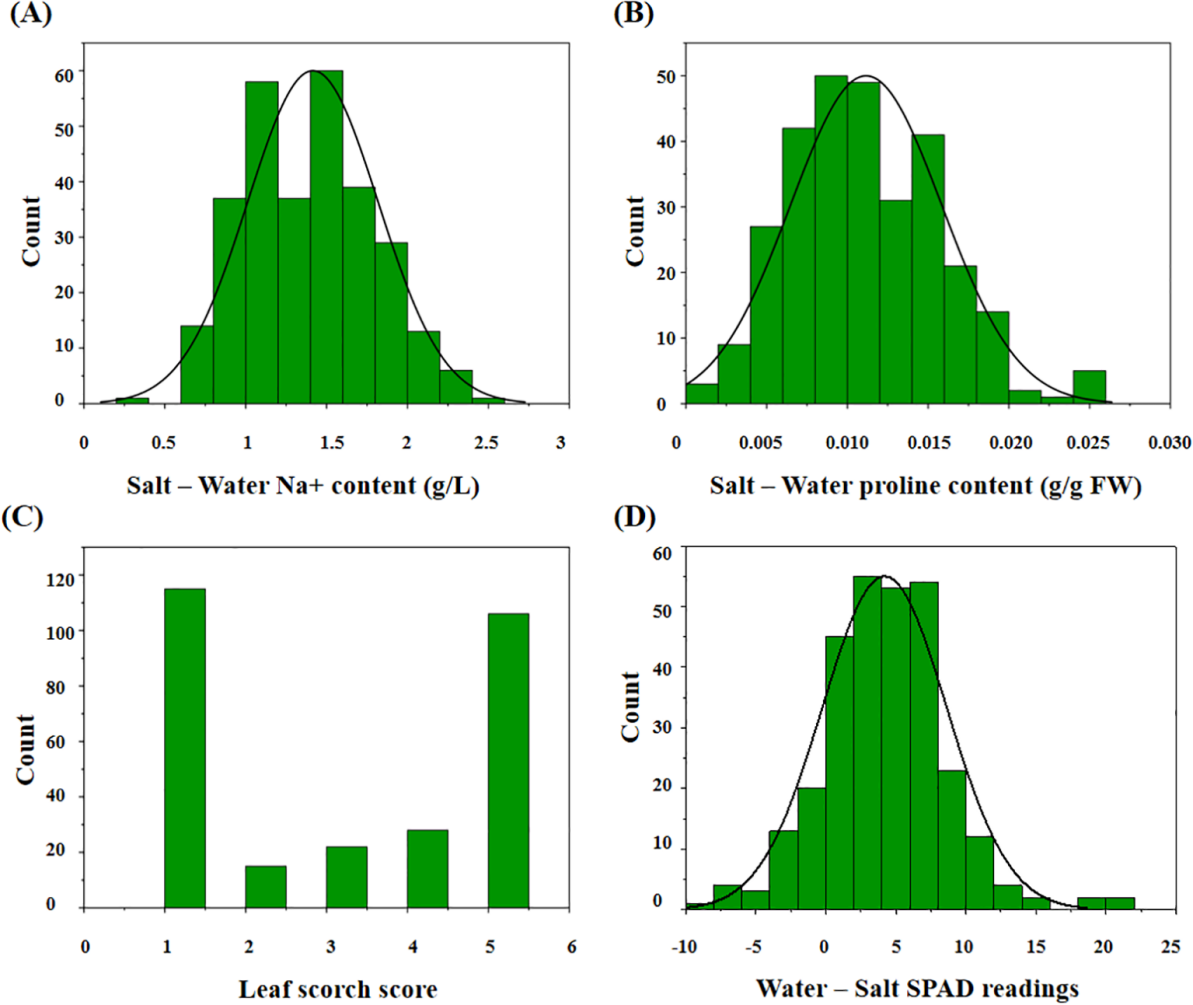
The distribution of the related traits after salt treatment in 295 peanut genotypes. **(A)** The change value of ion sodium content in the treatment group compared with the control group; **(B)** the change value of proline content in the treatment group compared with the control group; **(C)** the leaf scorch score of the treatment group; **(D)** the change value of leaf chlorophyll content in the treatment group compared with the control group.

Our results indicated that the change in sodium ion content between the treatment and control groups ranged from 0.30 to 2.48 g/L (average: 1.41 g/L; **Figure 2A**). The change in proline content ranged from 0.0014 to 0.0259 g/g FW (average: 0.0112 g/g FW; **Figure 2B**). The average LSS in the treatment group was 2.98 on a 1–5 scale (**Figure 2C**). The change in leaf chlorophyll content ranged from -8.55 to 21.37 (average: 4.19; **Figure 2D**). Traits associated with salt stress followed a normal distribution (**Supplementary Table S2**).

To examine the major contributors to phenotypic variation under salt stress, principal component analysis was performed using the prcomp function in R software; confidence ellipses were generated using the factoextra package (Kassambara and Mundt, 2016) in R software. The optimal number of clusters (K) was determined using the K-means clustering algorithm and the delta K (ΔK) method, testing K values from 1 to 15. The optimal K was identified as 2 (**Supplementary Figure 1A**). Our analysis divided the 295 peanut genotypes into two distinct groups based on phenotypic variation, representing the characteristics of salt-tolerant and salt-susceptible types, respectively. However, because the evaluated phenotypic traits are quantitative in nature, intermediate responses can also be considered within this variation. (**Supplementary Figure 1B**).

**Figure 3:**
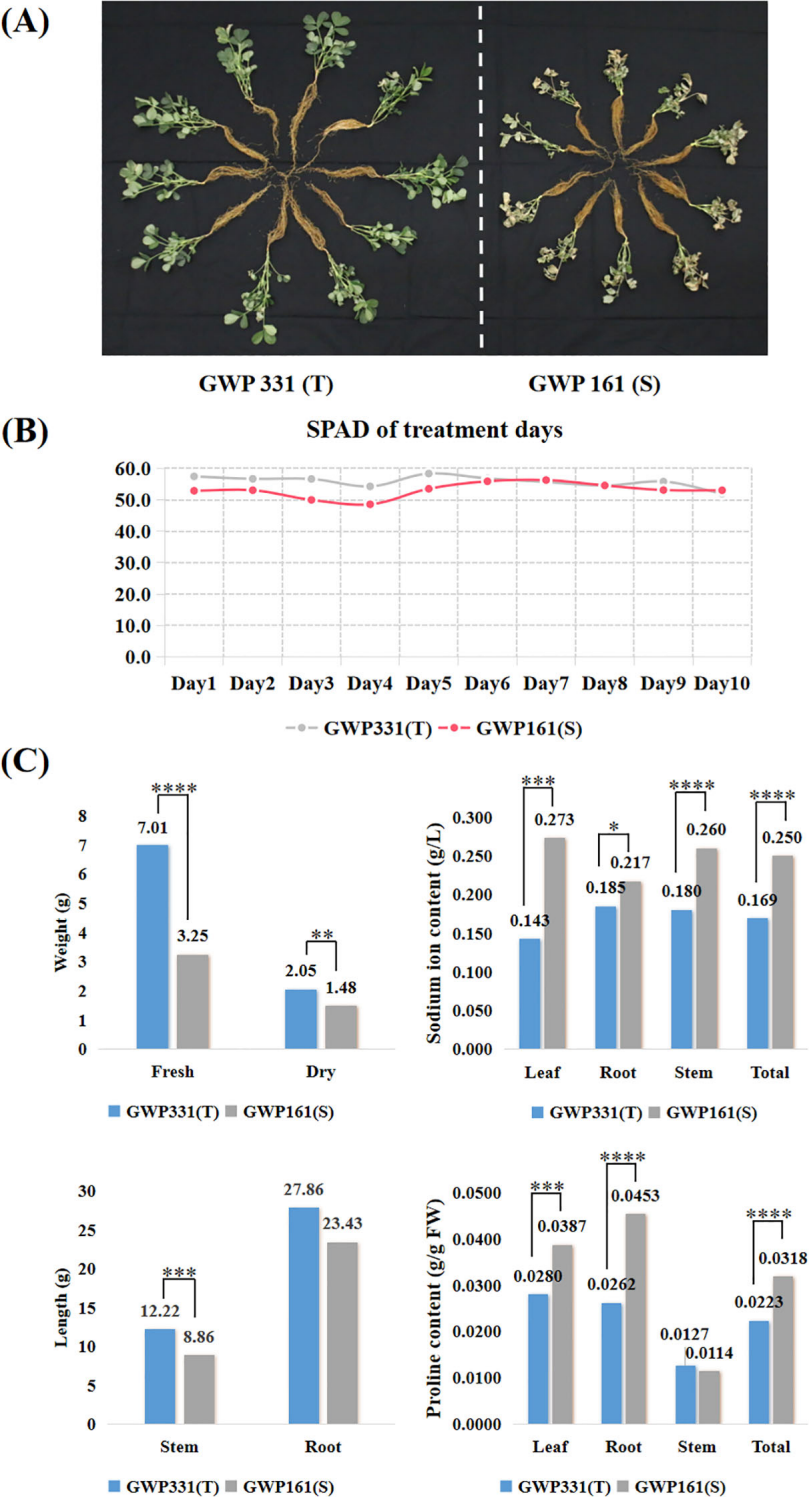
The phenotypes of two peanut genotypes of salt tolerance exposed to a 200 mM NaCl solution for ten days. **(A)** Plant phenotypic status after ten days of salt treatment; **(B)** SPAD for two peanut genotypes exposed to 200 mM NaCl for ten days; **(C)** Weight, length, sodium ion and proline accumulation for two peanut genotypes exposed to 200 mM NaCl for ten days. T-test p-values indicate statistical significance (*p < 0.05,**p < 0.01,***p < 0.001,****p < 0.0001).

For RNA-seq analysis, two peanut genotypes were treated with 200 mM NaCl for 10 days to induce salt stress (**Figure 3A**). There was no significant difference in chlorophyll content in the first five days after treatment, but significant differences began to appear from the sixth day (**Figures 3B**; **Supplementary Table S3**). The salt-susceptible genotype exhibited higher sodium ion and proline contents than the salt-tolerant genotype. Sodium ion concentrations in T and S were 1.69 g/L and 2.50 g/L, respectively. Proline concentrations were 0.0223 g/g FW in T and 0.0318 g/g FW in S (**Figures 3C**; **Supplementary Table S3**). Phenotypic data were tested for normality using SPSS 15.0. The Wilcoxon rank-sum test was used to assess statistical significance, and boxplots were generated using Origin software. All reported differences were statistically significant (p < 0.05).

### 3.2 Genome-wide association study

Among the 58,000 SNPs, 47,837 exhibited polymorphisms. After the removal of SNPs with high levels of missing data (> 20%), heterozygosity (> 20%), or low minor allele frequency (< 0.01), 21,014 SNPs were retained for association analysis. Of these, 9,600 and 11,414 SNPs were derived from subgenomes A and B, respectively (**Supplementary Figure 2A**). Although most filtered SNPs were evenly distributed across the chromosomes, several chromosomes exhibited relatively large gaps between SNPs (**Supplementary Figure 2B**).

The population structure of the 295 peanut genotypes was assessed using ADMIXTURE v1.3.0, and results were visualized with the pophelper package in R software. Structures for various parameters were compared using a cross-validation procedure with K values ranging from 1 to 15. The delta K (ΔK) method identified K=3 as the optimal number of clusters (**Supplementary Figure 2D**). The results indicated minimal genetic differentiation among peanut genotypes, including landraces, breeding lines, and cultivars, although evidence of genome introgression from genotypes of other origins was present (**Supplementary Figure 2D**).

**Figure 4:**
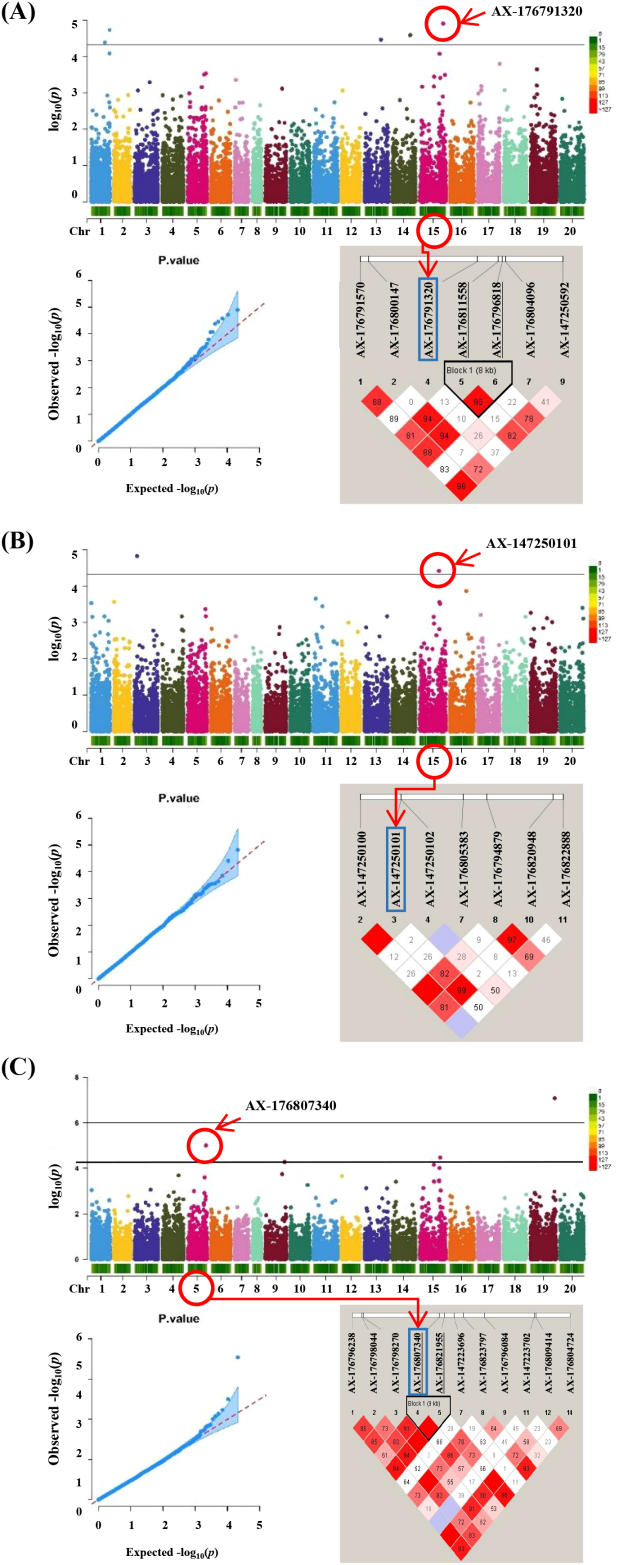
GWAS results and linkage disequilibrium (LD) plot related to salt stress traits in peanut. **(A)** Sodium ion content Manhattan plots followed by q-q plots and LD plot, **(B)** proline content Manhattan plots followed by q-q plots and LD plot and **(C)** leaf scorch score Manhattan plots followed by q-q plots and LD plot. Linkage disequilibrium (LD) plot showing r^2^ values within diamonds for selected SNPs, significant combined p values are highlighted in red.

The GWAS revealed associations among three salt tolerance–related traits in peanut (**Figure 4**). The observed and expected distributions of SNPs in the Q-Q plot demonstrated that population structure and kinship were clearly explained in the model. Significant SNPs were identified based on a Bonferroni-corrected p-value threshold of 4.74 × 10^−6^. Five SNPs—AX-176791320, AX-176794398, AX-176816867, AX-176806272, and AX-176801457—showed significant associations with sodium ion content and were located on four chromosomes. For proline content, two significant SNPs, AX-176813560 and AX-147250101, were identified on chromosomes Aradu.A03 and Araip.B05, respectively. Three SNPs associated with LSS were mapped to chromosomes Aradu.A05, Araip.B05, and Araip.B09 (**Table 1**). For chlorophyll content, no significant SNPs were detected in the study.

**Table 1:** The significant SNPs associated with three salt stress-related traits.

Trait	Marker	Chromosome	Position (bp)^b^	P.value
LSS^a^	AX-177641536	Araip.B09	139967796	8.40E-08
	AX-176807340	Aradu.A05	104179970	1.00E-05
	AX-176820297	Araip.B05	118278277	3.43E-05
Ion sodium content	AX-176791320	Araip.B05	134608088	1.36E-05
	AX-176794398	Aradu.A01	104994690	1.98E-05
	AX-176816867	Araip.B04	102685702	2.65E-05
	AX-176806272	Araip.B03	93368121	3.67E-05
	AX-176801457	Aradu.A01	77900866	4.06E-05
Proline content	AX-176813560	Aradu.A03	14798368	1.52E-05
	AX-147250101	Araip.B05	111647407	3.89E-05

a Leaf scorch score.

b the reference sequence come from the PEANUTBASE website tool (https://www.peanutbase.org).

To estimate LD decay in the population, pairwise comparisons of all SNPs were conducted. At a cutoff value of *r*^2^=0.1, the average LD decay across the 295 peanut genotypes was approximately 150 kb (**Supplementary Figure 2C**). The LD pattern across the genome revealed several haplotype blocks containing SNPs, which can be used to define the genomic range of candidate genes (**Figure 4**). Genomic regions containing significant SNPs identified via GWAS were examined to identify candidate genes based on the *A. hypogaea* Tifrunner 1.0 reference genome. Within ± 500 kb of the nine significant SNPs, 559 annotated genes were identified using the NCBI reference genome (**Supplementary Table S4**). The analysis revealed that the QTL regions on Arahy.01 and Arahy.15 contained the highest numbers of candidate genes, with 101 and 136 genes, respectively. In contrast, the fewest candidate genes were located within the Arahy.04 QTL region with 33 genes. Additionally, candidate genes were also identified on chromosomes Arahy.03, Arahy.05, Arahy.16, and Arahy.19.

**Figure 5:**
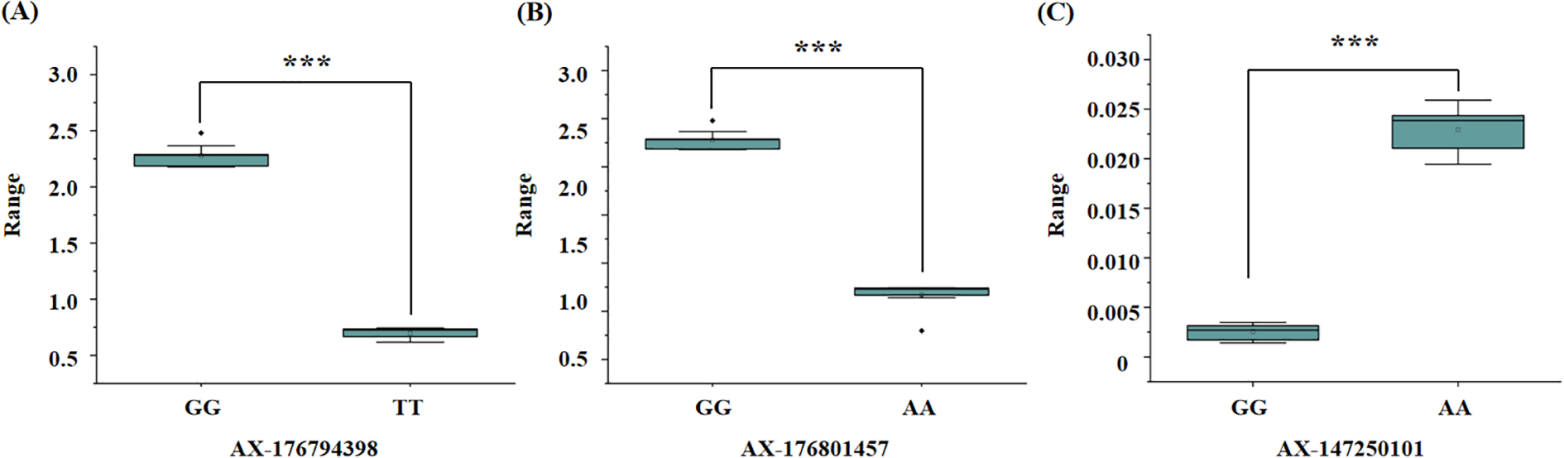
Boxplot of significant marker related to salt stress traits in peanut. **(A)** significant marker about the SNP AX-176794398, TT genotypes is salt tolerant samples and GG genotypes is salt susceptible samples; **(B)** significant marker about the SNP AX-176801457, AA genotypes is salt tolerant samples and GG genotypes is salt susceptible samples; **(C)** significant marker about the SNP AX-147250101, GG genotypes is salt tolerant samples and AA genotypes is salt susceptible samples. T-test p-values indicate statistical significance (*** p < 0.001).

To evaluate the genotypic frequencies of significant SNPs, 10 peanut genotypes with the highest values and 10 peanut genotypes with the lowest values for the three salt stress–related traits were selected. The SNPs exhibited statistically significant differences between groups, as determined by t-tests (p < 0.05) (**Figure 5**). Three SNPs—AX-176794398, AX-176801457, and AX-147250101—demonstrated strong associations with target traits and were selected as candidates for marker-assisted selection.

### 3.3 RNA-seq for salt stress tolerance

The total number of clean reads generated from each sample in the RNA-seq analysis was 120,169,372,770 bp (average read length: 101 bp). During preprocessing, bases with a Phred quality score (Q) below 30 were trimmed to ensure high-quality transcriptome data. After filtering, 1,189,795,770 bp (95.59% of bases with Q ≥ 30) were uniquely mapped, covering 65,763 gene annotations (**Supplementary Table S5**).

**Figure 6:**
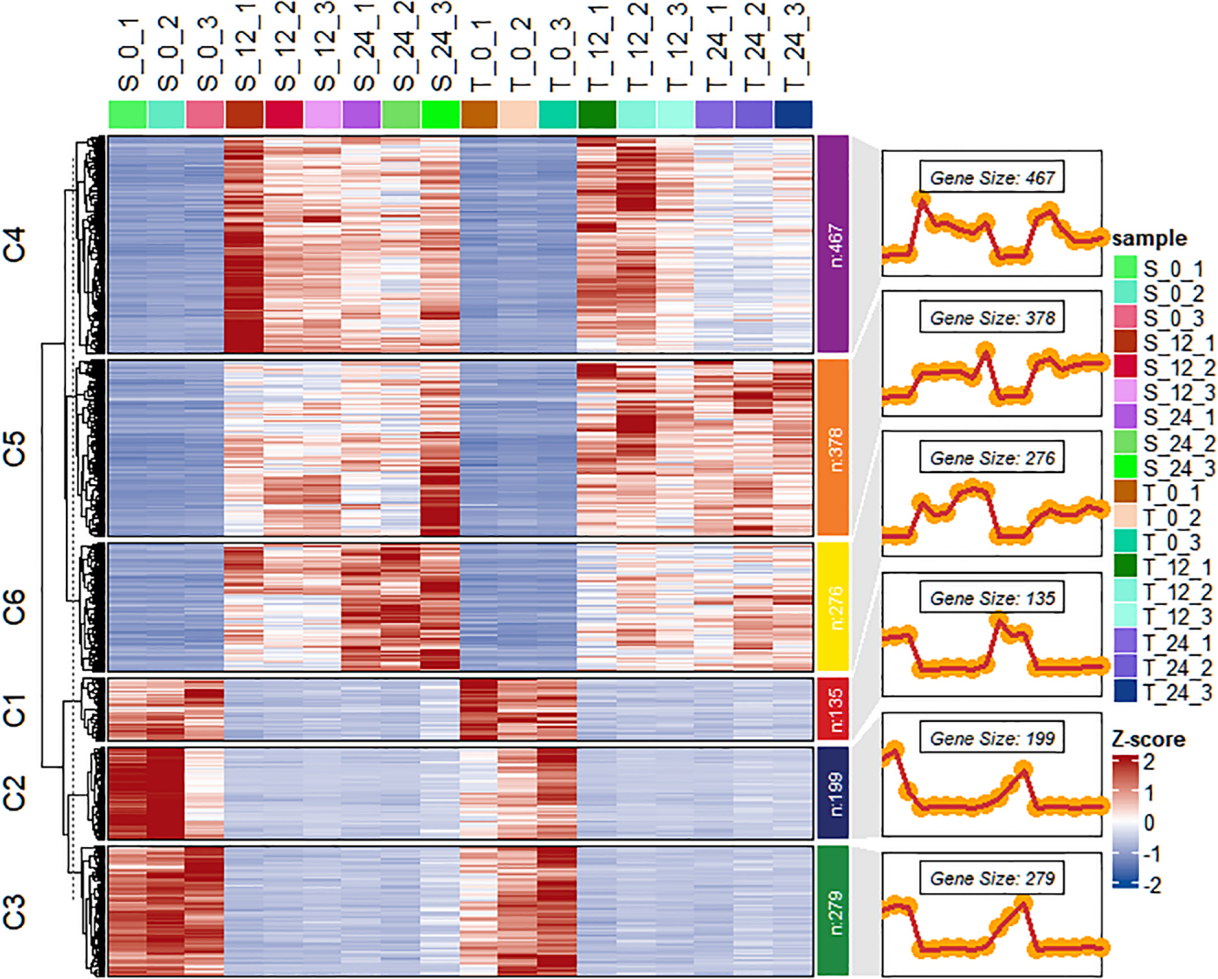
Heat map of the differential expression level of the genes in each cluster and line plots displaying the expressed clusters as patterns. T is GWP331 (salt-tolerant genotype), S is GWP161 (salt-sensitive genotype).

The DESeq2 package was used to perform functional analysis of DEGs across transcriptome groups. After salt stress treatment, 1,734 DEGs were identified between the T and S. Among these DEGs, 1,121 were specifically upregulated and 613 were specifically downregulated (**Supplementary Figure 3**). Hierarchical clustering analysis was carried out to validate gene expression patterns based on the 1,734 DEGs significantly expressed in the salt-tolerant genotype. These DEGs were grouped into six clusters, containing 135, 199, 279, 467, 378, and 276 genes, respectively (**Figure 6**). Clusters C1, C2, and C3 primarily contained downregulated genes, whereas clusters C4, C5, and C6 included genes that were predominantly upregulated in both genotypes. When expression was analyzed across time points, downregulated genes exhibited similar patterns in both genotypes. However, the upregulated clusters demonstrated distinct expression differences between salt-tolerant and salt-susceptible lines.

To explore the functional roles of these DEGs, GO enrichment analysis and KEGG pathway analysis were conducted for each of the six gene clusters. Overall, the 1,734 DEGs were enriched across 126 GO terms (**Supplementary Table S6**, **Supplementary Figure 4A**), including 55 terms classified under molecular function, 64 under biological process, and seven under cellular component. In the molecular function category, DEGs were predominantly associated with oxidoreductase activity and catalytic activity. In the biological process category, genes were enriched in processes such as biosynthesis and defense responses. KEGG pathway analysis revealed that the 1,734 DEGs were involved in 60 pathways (**Supplementary Figure 4B**). These pathways included, but were not limited to, flavonoid biosynthesis, transcription factor regulation, cytochrome P450 pathways, and various signal transduction pathways. Candidate genes closely associated with salt stress were identified through integration of hub gene analysis within the gene co-expression network (**Supplementary Figure 5**) and their corresponding GO annotations. GO and KEGG enrichment analyses were conducted for genes within the interaction network, revealing enrichment across 298 GO terms. These terms included categories such as response to stimulus, response to stress, oxidation–reduction processes, catalytic activity, oxidoreductase activity, and membrane-associated functions. Comparison of these genes also indicated significant enrichment in three KEGG pathways: metabolic pathways, plant hormone signal transduction, and the mitogen-activated protein kinase signaling pathway, as well as valine, leucine, and isoleucine degradation pathways.

### 3.4 Identification of common genes between GWAS and RNA-seq analyses

Seventeen common candidate genes associated with the three salt stress–related traits were identified through comparative analysis of results from GWAS and RNA-seq (**Table 2**).

**Table 2:** Functional annotations for 17 common genes with significant expression patterns.

Trait	Marker	Gene name	Position	Annotation
Ion sodium content	AX-176794398	LOC112709782	Arahy.01:110360091-110362687	UDP-glucose 6-dehydrogenase 3
		LOC112709906	Arahy.01:110507125-110511157	probable LRR receptor-like serine/threonine-protein kinase At1g74360
	AX-176801457	LOC112710495^b^	Arahy.01:111707918-111714684	delta-1-pyrroline-5-carboxylate dehydrogenase 12A1, mitochondrial
		LOC112710599^b c^	Arahy.01:111821960-111823694	uncharacterized LOC112710599
		LOC112710672^b c^	Arahy.01:112200136-112210940	zinc finger protein BRUTUS-like At1g18910
	AX-176816867	LOC112797053^b c^	Arahy.04:100532219-100533986	malonyl-CoA:anthocyanidin 5-O-glucoside-6’’-O-malonyltransferase
		LOC112797068	Arahy.04:101530682-101536114	ammonium transporter 2
	AX-176806272	LOC112755811	Arahy.16:148806585-148810393	7-deoxyloganetin glucosyltransferase
		LOC112755817	Arahy.16:148884763-148886792	NAD(P)H-dependent 6’-deoxychalcone synthase
		LOC112755872	Arahy.16:149443901-149446350	protein ALP1-like
Proline content	AX-176813560	LOC112789620	Arahy.03:14207411-14211820	transcription factor CPC
	AX-147250101	LOC112750538	Arahy.15:120856002-120858287	allene oxide cyclase, chloroplastic
LSS^a^	AX-176807340	LOC112800558^b c^	Arahy.05:7823605-7824645	ethylene-responsive transcription factor ERF011
		LOC112800595^b^	Arahy.05:8637995-8640483	patatin-like protein 1
	AX-176820297	LOC112750632^b^	Arahy.15:128041572-128047127	protein NUCLEAR FUSION DEFECTIVE 4
	AX-177641536	LOC112777181	Arahy.19:152066529-152069467	ABC transporter G family member 6
		LOC112777442	Arahy.19:152107029-152109194	chalcone–flavonone isomerase 1A

a Leaf scorch score.

b Candidate genes showing significant difference based on gene expression analysis using qRT-PCR.

c the reference sequence come from *A. hypogaea* Tifrunner 1.0 reference genome of National Center for Biotechnology Information (NCBI).

**Figure 7:**
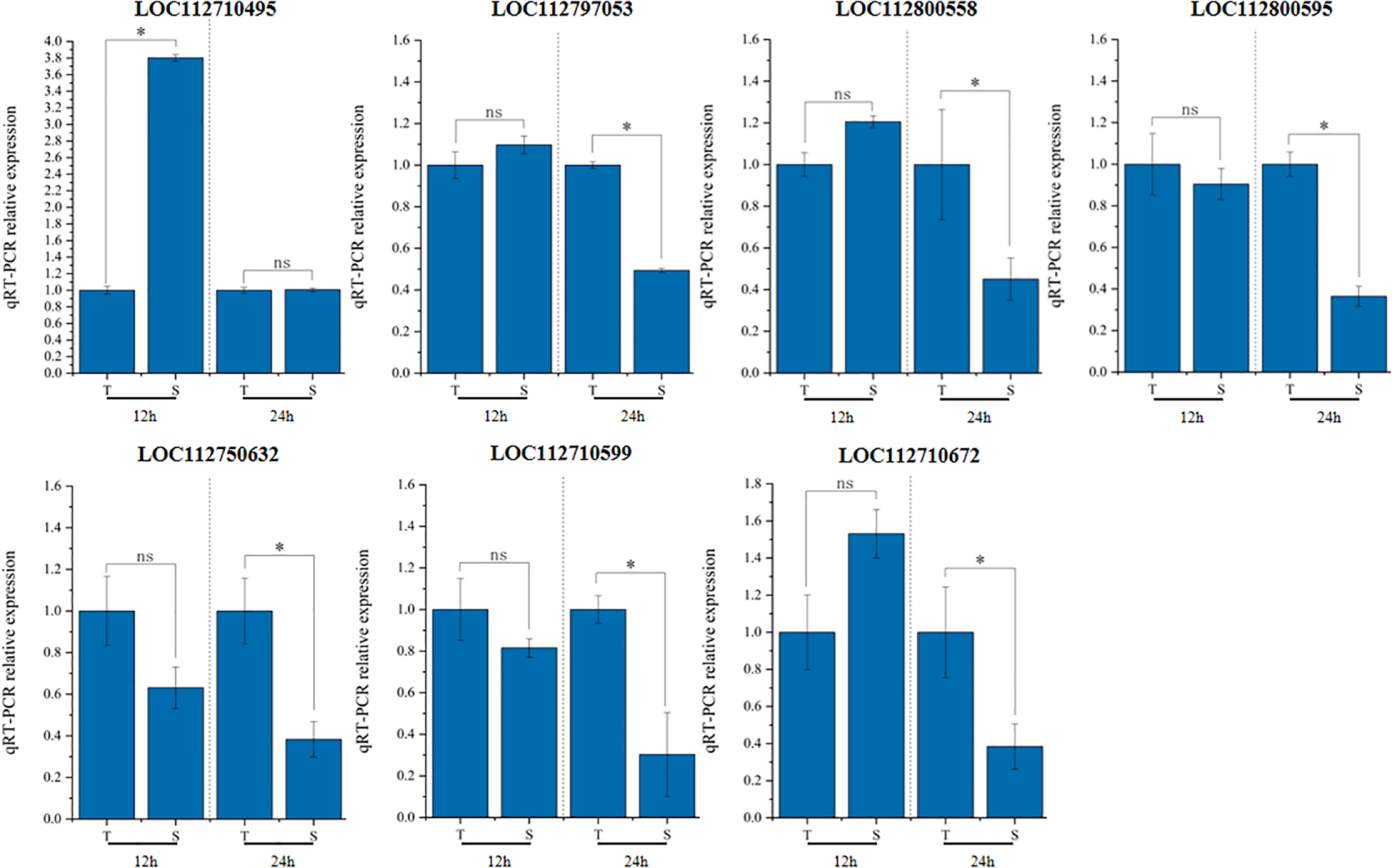
Gene expression analysis by qRT-PCR. qRT-PCR result based on root tissues after treatment for 12h and 24h. T is GWP331 (salt-tolerant genotype), S is GWP161 (salt-sensitive genotype). *Represents a significant difference at least more than two-fold. ^ns^not significant.

Expression levels of these 17 genes were initially examined in T and S. Among them, four genes (*LOC112710495*, *LOC112710599*, *LOC112710672*, and *LOC112797053*) associated with sodium ion content, and three genes (*LOC112800558*, *LOC112800595*, and *LOC112750632*) associated with LSS, showed significant expression differences between the two genotypes at least at two time points (**Figure 7**). The remaining 10 genes did not exhibit statistically significant differences in expression.

The seven candidate genes with significant expression differences were sequenced in T and S. Promoter regions, coding exons, and exon–intron junctions were amplified by PCR, then subjected to direct DNA sequencing in both forward and reverse directions (**Supplementary Figure 6**). Sequence analysis revealed mutations in two genes associated with sodium ion content and LSS. The *LOC112797053* gene, linked to sodium ion content, contained a base substitution in the promoter region and a synonymous SNP in the exon, along with an adjacent SNP on chromosome Arahy.04. Additionally, analysis of *LOC112750632* revealed a single SNP in the promoter region on chromosome Arahy.15. These two genes encode malonyl-CoA:anthocyanidin 5-O-glucoside-6’’-O-malonyltransferase and NUCLEAR FUSION DEFECTIVE 4, respectively.

### 3.5 Development of KASP markers

**Figure 8:**
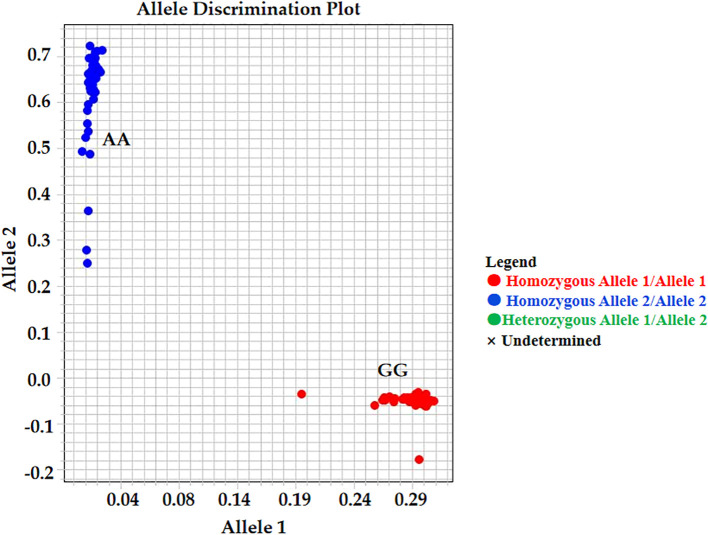
Kompetitive allele-specific PCR (KASP) analysis result. KASP genotypes about the SNP AX-147250101, GG genotypes is salt tolerant samples and AA genotypes is salt susceptible samples.

Based on the validated mutation information, the KASP marker AX-147250101 was developed. The genotypes identified by this marker included four categories: homozygous resistant, homozygous susceptible, heterozygous, and missing. For AX-147250101 (**Figure 8**; **Supplementary Table S7**), 6.7% of accessions in the group with the lowest proline content were homozygous resistant, whereas 93.3% were homozygous susceptible. In contrast, among accessions with the highest proline content, 96.7% were homozygous resistant and 3.3% were homozygous susceptible. These results indicate that the AX-147250101 KASP marker can effectively differentiate proline content among peanut accessions and can be used to identify salt-tolerant peanut varieties.

## 4 Discussion

Excessive soil salinity is a major abiotic stress that adversely affects crop productivity and contributes to global economic losses (Shrivastava and Kumar, 2015). Salt-tolerant crop varieties are needed to mitigate this issue. Although most crops exhibit susceptibility to elevated salt concentrations, responses vary among crop species, varieties, and developmental stages (Di Gioia et al., 2018). Soil salinity levels between 2 and 4 dS m^−1^ can reduce yield in salt-sensitive plants, and levels above 8 dS m^−1^ severely impair growth in most crops (Hillel, 2000). Multiple studies have evaluated salinity tolerance in various crop species, including wild rice (Quan et al., 2018), tomato (Guo et al., 2022), and soybean (Do et al., 2019). Peanuts are generally considered moderately susceptible to salt stress (Cui et al., 2018). There is evidence that reduction of the transcription factor ABSCISIC ACID INSENSITIVE 4s enhances salt tolerance and biomass accumulation in peanut seedlings under saline conditions (Luo et al., 2021). However, few studies have focused on the genetic basis of salt stress tolerance in peanut. Therefore, comprehensive investigation of salt tolerance–related genes, selection of salt-tolerant genotypes, and development of salt-tolerant peanut cultivars are critical priorities for current and future peanut breeding programs.

Aboveground tissues, particularly leaves, often show more pronounced differences between salt-resistant and salt-sensitive cultivars due to the regulated transport of sodium ions from roots to shoots, and indeed many previous studies have focused on leaf tissues. In our study, we made the deliberate choice to focus on root tissues based on our preliminary physiological data (**Figure 3**), which revealed significant differences in sodium ion and proline accumulation in peanut roots, as well as in aboveground tissues, following salt stress treatment. Since roots serve as the primary interface for ion uptake and the initial site of salt perception, we hypothesized that critical regulatory mechanisms governing salt tolerance might be operating at this entry point. Furthermore, several recent studies on peanut salt tolerance have successfully utilized root tissues for transcriptomic analysis, demonstrating the validity of this approach (Chen et al., 2016a; Wang et al., 2024). In future studies incorporating leaf, root and shoot tissues at multiple time points would provide a more comprehensive understanding of peanut’s salt tolerance mechanisms.

The 58,000 SNP genotypic data from the Axiom_Arachis array have played a key role in advancing the understanding of the evolutionary and domestication history of peanut (Bertioli et al., 2019). The application of this array has also demonstrated its utility and reliability in screening peanut genotype resources with diverse genetic backgrounds. Our investigation into salt tolerance–related genes in peanut was greatly aided by the availability of the publicly accessible reference genome and the high-density SNP array. The estimated genome size of cultivated peanut is approximately 2.54 Gb (Zhuang et al., 2019). In this study, we utilized a 2.27 Gb high-quality genome assembly covering chromosomes A01–A10 (A subgenome) and B01–B10 (B subgenome). Based on an LD decay distance of approximately 150–200 kb, the number of markers required for effective GWAS ranges from 12,700 to 17,000. This is fewer than the 21,014 SNPs used in the present study, thus emphasizing the adequacy and density of our marker set. False positives resulting from population structure and relatedness among accessions represent a major concern in GWAS. To address this concern, mixed linear model approaches have been developed to reduce such errors (Kaler and Purcell, 2019; Yu et al., 2006). Accordingly, we implemented the enriched compressed mixed linear model in this study to identify significant associations between SNPs and phenotypic traits of interest.

Recent studies have demonstrated the effectiveness of integrating GWAS and RNA-seq to identify genes associated with salt stress tolerance, a critical trait for many crops cultivated in increasingly saline soils. In barley, for example, this combined approach identified 54 SNPs associated with salt tolerance, along with several salt-responsive genes involved in ion homeostasis, antioxidant activity, and osmotic regulation—key functions that support plant adaptation to salt stress (Xu et al., 2023b). In maize, system-level integration of stress response pathways revealed key functional candidates, particularly those associated with protein–protein interactions and flavonoid metabolic processes, which play important roles in high pH tolerance (Li et al., 2022). In mung bean, this approach enabled the identification of candidate genes linked to germination rates under salt stress, highlighting its utility in uncovering molecular mechanisms of salt and alkaline stress tolerance (Xu et al., 2023a). The parallel application of GWAS and RNA-seq offers substantial advantages. GWAS identifies SNPs associated with traits related to salt tolerance, thus revealing potential genetic markers. By providing gene expression profiles, RNA-seq allows direct observation of candidate gene responses under salt stress. Generally, GWAS identify association signals within broad genomic intervals, often spanning hundreds of kilobases and encompassing multiple candidate genes. Unlike previous studies that detected large genomic regions, our integrated GWAS and RNA-seq approach localized candidate genes within 100–200 kb windows, thereby improving mapping precision. By cross-validating association regions with gene expression profiles, we effectively narrowed down the candidate intervals and enhanced the reliability of gene identification (Chen et al., 2025; Lv et al., 2024). Therefore, compared with earlier single-approach studies, this integrative framework provides greater resolution for functional gene discovery and strengthens the potential application of these findings in molecular breeding programs for peanut.

Multiple studies have examined the effects of salt stress in peanut; however, the protein interactions and functional mechanisms underlying salt tolerance in peanut remain unclear. For example, Xu et al. (2022) conducted a comprehensive evaluation of salt stress impacts by analyzing cultivar-specific responses in rhizosphere bacterial community diversity, plant morphology, and pod yield. Their study combined statistical analysis with 16S rRNA gene sequencing to clarify these relationships. The results demonstrated that salt stress significantly affected peanut growth and pod yield; different cultivars exhibited distinct responses. Another study provided transcriptomic and physiological evidence concerning the association between unsaturated fatty acids and salt stress in peanut (Sui et al., 2018). It also identified 178 protein phosphatase 2C (PP2C) genes unevenly distributed across the 20 chromosomes, which appeared to play important roles in salt stress response by influencing metabolic processes, hormone levels, and growth regulators. These findings offered additional insights into the PP2C gene family in peanut and suggested that PP2C genes are involved in the plant’s response to salt stress (Wu et al., 2023). Protein–protein interactions play essential roles in many biological processes. Protein–protein interaction networks derived from omics data constitute valuable resources for elucidation of mechanisms that underlie biological functions and prediction of protein activity. In the context of salt stress in peanut, protein–protein interaction networks help describe the roles of proteins corresponding to DEGs and aid in understanding intracellular response mechanisms under saline conditions. The present study identified DEGs associated with salt stress at the transcriptome level in peanut (**Figure 6**). High-throughput data and pathway-based predictions offer guidance for future research, particularly in identifying novel proteins and phosphorylation sites involved in salt stress. Such findings may support further investigations into the regulation of ion homeostasis in peanuts under salt stress conditions.

Two candidate genes exhibiting mutations potentially associated with salt tolerance in peanut were identified in this study (**Table 2**, **Supplementary Figure 6**). No previous reports have described a relationship between these genes and salt stress. Anthocyanins, a class of plant flavonoids, are often modified by propylene glycol groups; however, the enzyme responsible for catalyzing the malondialdehyde reaction has not been clearly identified. Phylogenetic analyses have shown that fatty acylation and aromatic acylation of anthocyanins are typically catalyzed by members of the plant acyltransferase family, particularly those in specific subfamilies (Suzuki et al., 2001). In *Arabidopsis thaliana*, the major anthocyanin is a cyanidin derivative that undergoes glycosylation and acylation with malonyl, *p*-coumaroyl, and sinapoyl groups. The enzyme responsible for malonylation has been identified as a member of the BAHD acyltransferase family. The transcription level of the epistatic gene *At3g29590*, which encodes anthocyanin 5-O-glucoside-6″-O-malonyltransferase (At5MAT), increases in conjunction with anthocyanin pigment accumulation in certain plant strains. This enzyme, known as malonyltransferase, exhibits substrate specificity for malonyl-coenzyme A and 5-O-glycosylated anthocyanins (D’Auria et al., 2007). Anthocyanins act as effective antioxidants that alleviate reactive oxygen species (ROS) and contribute to osmotic regulation, thereby enhancing plant tolerance to abiotic stresses such as salt stress. These findings provide a theoretical basis for the potential role of the malonyltransferase gene in regulating ROS scavenging and osmotic balance through anthocyanin modification (Naing and Kim, 2021). The protein NUCLEAR FUSION DEFECTIVE 4 (NFD4), encoded by *AT1G31460* in *A. thaliana*, is essential for karyogamy during female gametophyte development; specifically, it facilitates the fusion of two polar nuclei to form the diploid central cell nucleus (Portereiko et al., 2006). In addition to its role in reproduction, NFD4 is potentially involved in responses to abiotic stresses (e.g., salt stress), suggesting broader functional relevance (https://www.ncbi.nlm.nih.gov/IEB/Research/Acembly/av.cgi?db=ara&F=NFD4andAT1G31460). GO annotations for biological processes related to NFD4 indicate its involvement in salt stress response (https://www.arabidopsis.org/servlets/TairObject?type=locus&name=At1g31470). This finding underscores the need for further investigation of these two candidate genes to determine their roles in salt tolerance or salt stress response in peanut. Future studies will focus on the development of transgenic plants to evaluate gene function and regulatory mechanisms under salt stress conditions. This approach will support elucidation of the process by which these genes contribute to physiological responses in salt-stressed environments. In parallel, protein analysis techniques will be utilized to investigate expression patterns, interaction networks, and functional properties of the corresponding proteins. Structural and functional characterization, along with interaction profiling, will help to understand the roles of these proteins in salt stress response. These analyses are expected to provide a strong theoretical foundation for further research concerning salt tolerance mechanisms in peanut and other crop species.

## 5 Conclusions

This study provides novel insights into the genetic basis underlying salt stress tolerance in peanut through the integration of GWAS and RNA-seq analyses. By integrating large-scale phenotypic screening of 295 genotypes with high-density SNP genotyping and transcriptome profiling, we identified seven common candidate genes whose expression levels were significantly correlated with key salt tolerance traits. Among these, LOC112797053 may play critical roles in regulating ROS scavenging, osmotic balance, and stress signaling. Furthermore, the development of the KASP marker AX-147250101 enables precise differentiation of salt-tolerant genotypes, providing a practical tool for marker-assisted breeding. The findings not only expand the current understanding of salt tolerance mechanisms in peanut but also offer a valuable genomic foundation for accelerating the development of resilient cultivars adapted to saline environments, contributing to sustainable peanut production under increasing soil salinization.

## Data Availability

The datasets generated for this study can be found in the [NABIC], [https://nabic.rda.go.kr/nolog/NN-8246-000001/ngsSraView.do]. [https://nabic.rda.go.kr/nolog/NN-8245-000001/ngsSraView.do]. [https://nabic.rda.go.kr/nolog/NN-8244-000001/ngsSraView.do]. [https://nabic.rda.go.kr/nolog/NN-8243-000001/ngsSraView.do]. [https://nabic.rda.go.kr/nolog/NN-8242-000001/ngsSraView.do]. [https://nabic.rda.go.kr/nolog/NN-8235-000001/ngsSraView.do].
