# Supplementary material for: Genome-wide association and RNA-seq analyses reveal genes linked to salt stress in peanut (Arachis hypogaea L.)
Source: Front Plant Sci. 2025 Nov 27;16:1699469. doi: 10.3389/fpls.2025.1699469 (PMC12695741; doi:10.3389/fpls.2025.1699469)
Supplement: Supplementary file 1 [file Presentation1.pptx]

## Slide 1
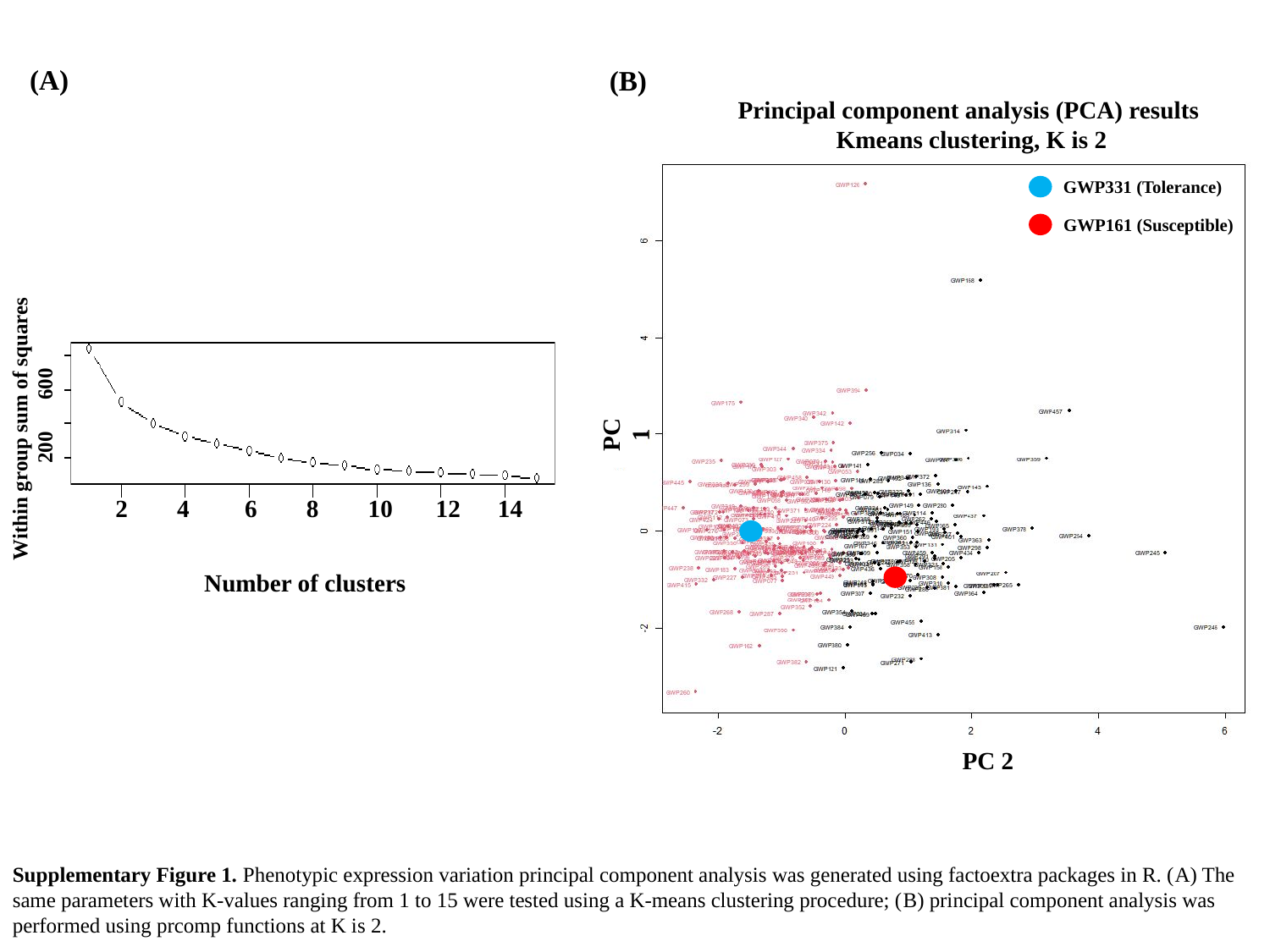

(A)
(B)
Principal component analysis (PCA) results
Kmeans clustering, K is 2
GWP331 (Tolerance)
GWP161 (Susceptible)
Within group sum of squares
200 600
PC 1
2 4 6 8 10 12 14
Number of clusters
PC 2
Supplementary Figure 1. Phenotypic expression variation principal component analysis was generated using factoextra packages in R. (A) The same parameters with K-values ranging from 1 to 15 were tested using a K-means clustering procedure; (B) principal component analysis was performed using prcomp functions at K is 2.
