# Supplementary material for: Genome-wide association and RNA-seq analyses reveal genes linked to salt stress in peanut (Arachis hypogaea L.)
Source: Front Plant Sci. 2025 Nov 27;16:1699469. doi: 10.3389/fpls.2025.1699469 (PMC12695741; doi:10.3389/fpls.2025.1699469)
Supplement: Supplementary file 2 [file Presentation2.pptx]

## Slide 1
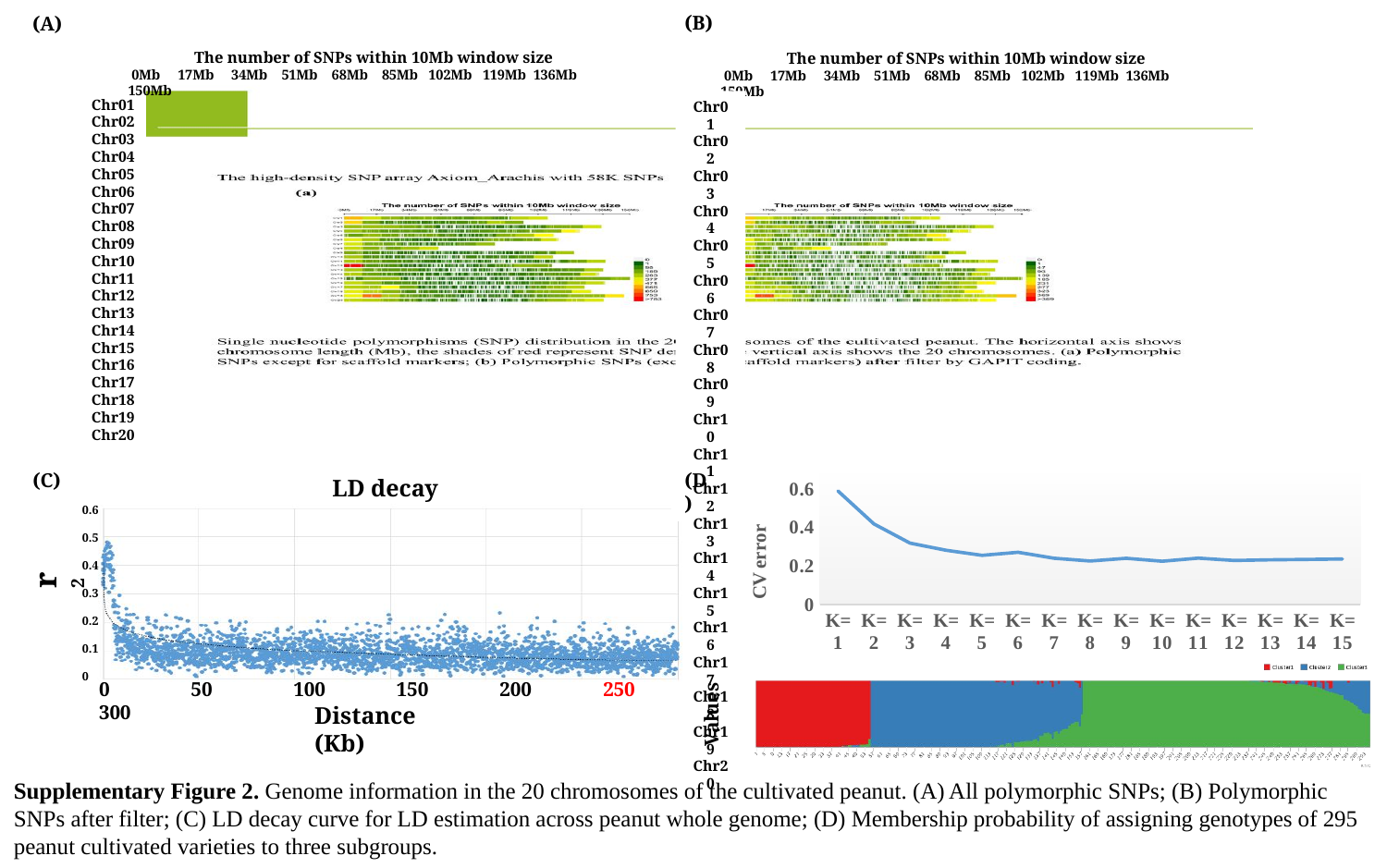

(B)
(A)
The number of SNPs within 10Mb window size
 0Mb 17Mb 34Mb 51Mb 68Mb 85Mb 102Mb 119Mb 136Mb 150Mb
The number of SNPs within 10Mb window size
 0Mb 17Mb 34Mb 51Mb 68Mb 85Mb 102Mb 119Mb 136Mb 150Mb
Chr01
Chr02
Chr03
Chr04
Chr05
Chr06
Chr07
Chr08
Chr09
Chr10
Chr11
Chr12
Chr13
Chr14
Chr15
Chr16
Chr17
Chr18
Chr19
Chr20
Chr01
Chr02
Chr03
Chr04
Chr05
Chr06
Chr07
Chr08
Chr09
Chr10
Chr11
Chr12
Chr13
Chr14
Chr15
Chr16
Chr17
Chr18
Chr19
Chr20
(D)
(C)
### Chart
| Category | |
|---|---|
| K=1 | 0.58946 |
| K=2 | 0.41824 |
| K=3 | 0.31903 |
| K=4 | 0.28207 |
| K=5 | 0.25575 |
| K=6 | 0.27159 |
| K=7 | 0.24075 |
| K=8 | 0.22608 |
| K=9 | 0.24033 |
| K=10 | 0.22516 |
| K=11 | 0.24134 |
| K=12 | 0.22915 |
| K=13 | 0.23239 |
| K=14 | 0.23432 |
| K=15 | 0.23655 |LD decay
0.6
0.5
0.4
0.3
0.2
0.1
0
r2
Values
0 50 100 150 200 250 300
Distance (Kb)
Supplementary Figure 2. Genome information in the 20 chromosomes of the cultivated peanut. (A) All polymorphic SNPs; (B) Polymorphic SNPs after filter; (C) LD decay curve for LD estimation across peanut whole genome; (D) Membership probability of assigning genotypes of 295 peanut cultivated varieties to three subgroups.
