# Supplementary material for: Genome-wide association and RNA-seq analyses reveal genes linked to salt stress in peanut (Arachis hypogaea L.)
Source: Front Plant Sci. 2025 Nov 27;16:1699469. doi: 10.3389/fpls.2025.1699469 (PMC12695741; doi:10.3389/fpls.2025.1699469)
Supplement: Supplementary file 3 [file Presentation3.pptx]

## Slide 1
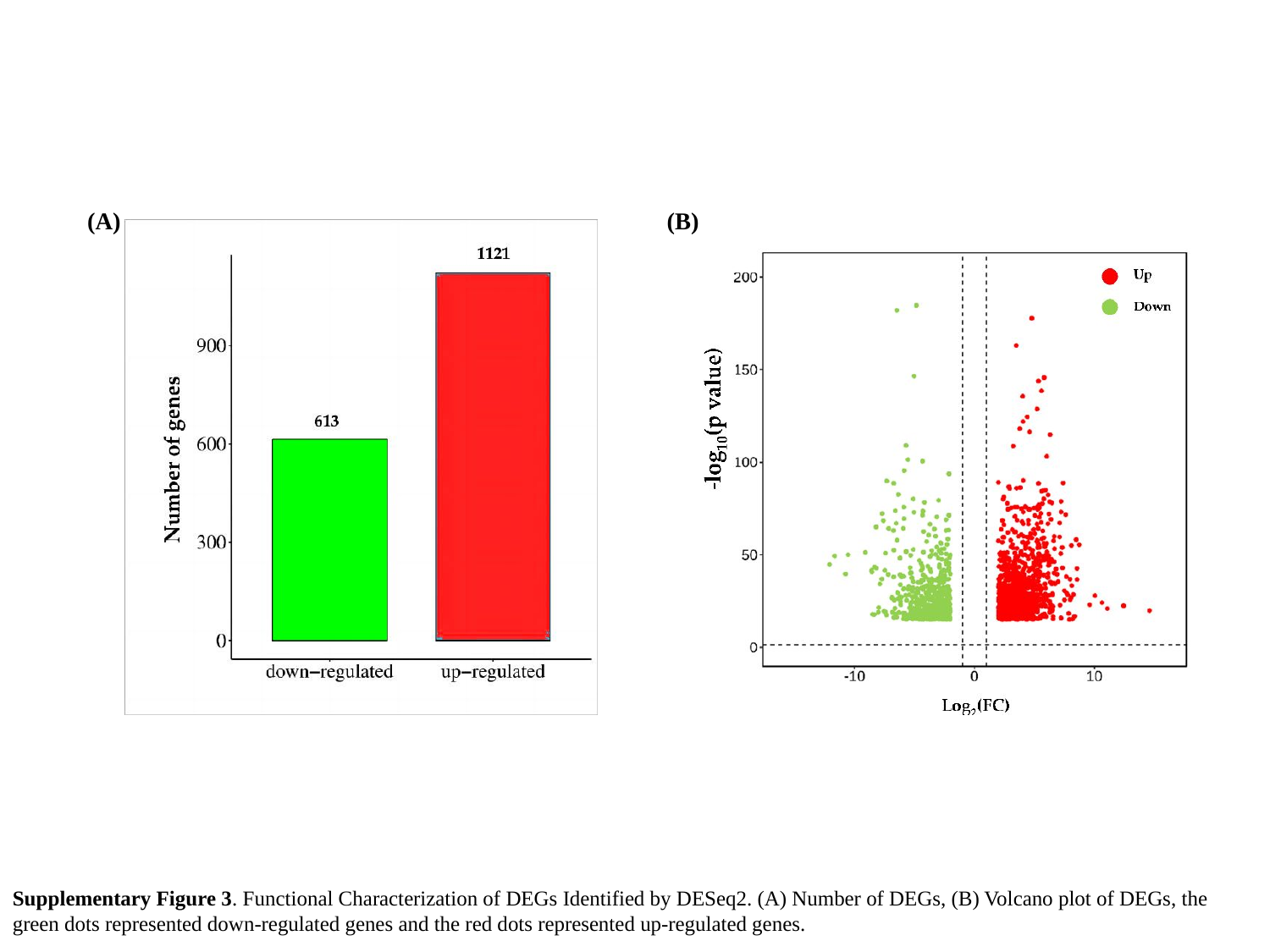

(A)
(B)
Supplementary Figure 3. Functional Characterization of DEGs Identified by DESeq2. (A) Number of DEGs, (B) Volcano plot of DEGs, the green dots represented down-regulated genes and the red dots represented up-regulated genes.
