# Supplementary material for: Genome-wide association and RNA-seq analyses reveal genes linked to salt stress in peanut (Arachis hypogaea L.)
Source: Front Plant Sci. 2025 Nov 27;16:1699469. doi: 10.3389/fpls.2025.1699469 (PMC12695741; doi:10.3389/fpls.2025.1699469)
Supplement: Supplementary file 4 [file Presentation4.pptx]

## Slide 1
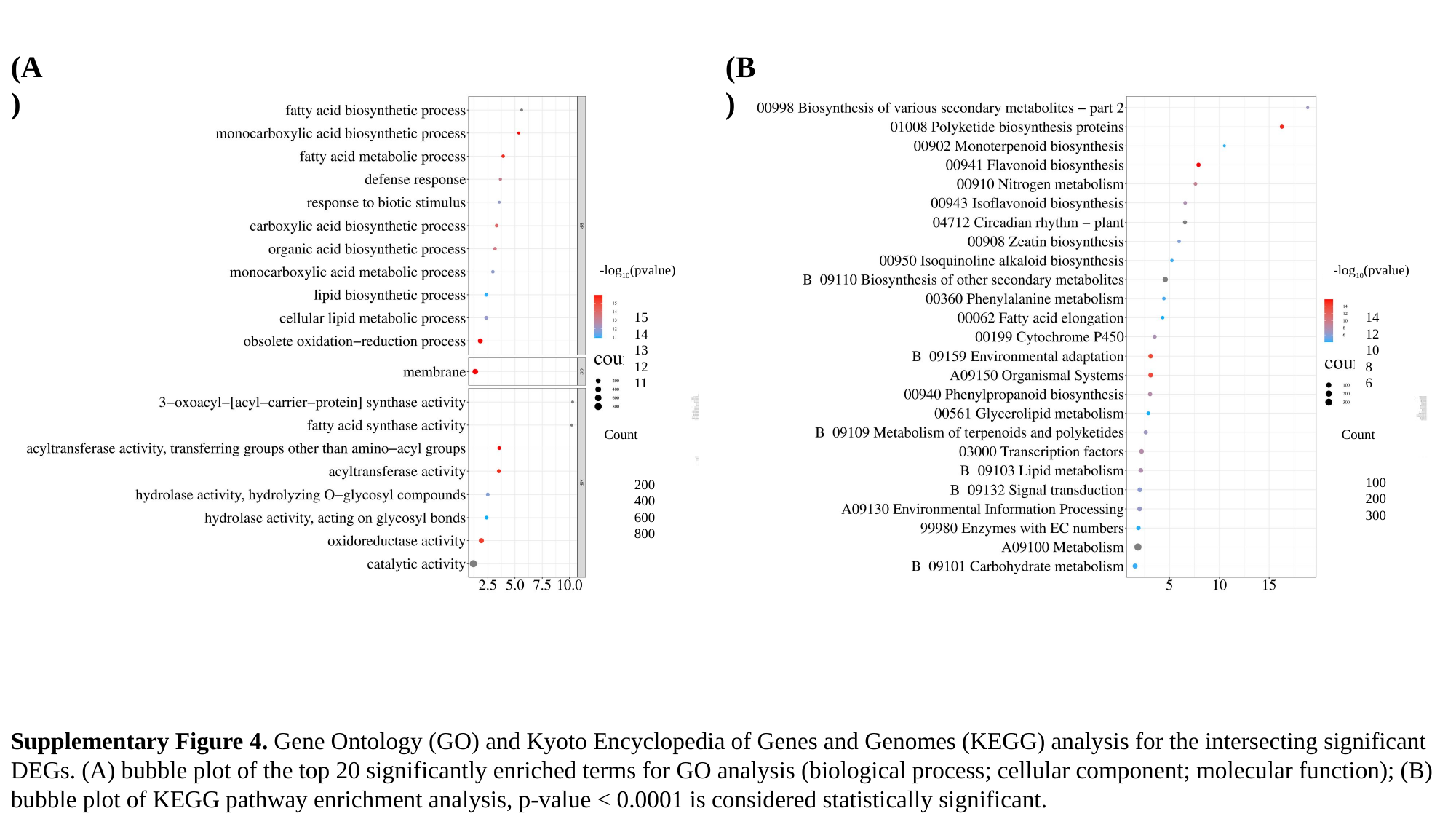

(A)
(B)
(B)
(A)
-log10(pvalue)
-log10(pvalue)
15
14
13
12
11
14
12
10
8
6
Count
Count
100
200
300
200
400
600
800
Supplementary Figure 4. Gene Ontology (GO) and Kyoto Encyclopedia of Genes and Genomes (KEGG) analysis for the intersecting significant DEGs. (A) bubble plot of the top 20 significantly enriched terms for GO analysis (biological process; cellular component; molecular function); (B) bubble plot of KEGG pathway enrichment analysis, p-value < 0.0001 is considered statistically significant.
