# Supplementary material for: Genome-wide association and RNA-seq analyses reveal genes linked to salt stress in peanut (Arachis hypogaea L.)
Source: Front Plant Sci. 2025 Nov 27;16:1699469. doi: 10.3389/fpls.2025.1699469 (PMC12695741; doi:10.3389/fpls.2025.1699469)
Supplement: Supplementary file 5 [file Presentation5.pptx]

## Slide 1
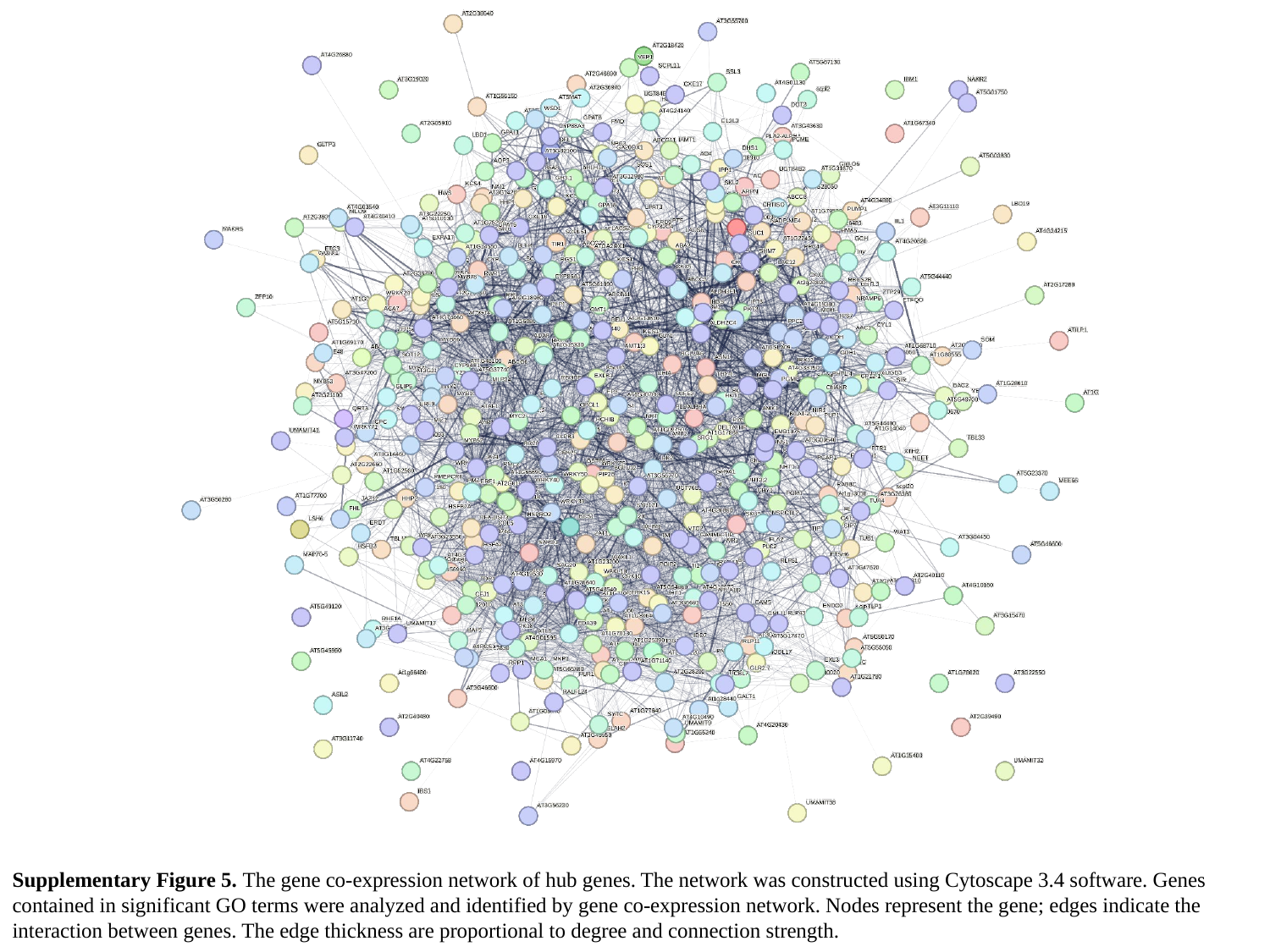

Supplementary Figure 5. The gene co-expression network of hub genes. The network was constructed using Cytoscape 3.4 software. Genes contained in significant GO terms were analyzed and identified by gene co-expression network. Nodes represent the gene; edges indicate the interaction between genes. The edge thickness are proportional to degree and connection strength.
