# Supplementary material for: Genome-wide association and RNA-seq analyses reveal genes linked to salt stress in peanut (Arachis hypogaea L.)
Source: Front Plant Sci. 2025 Nov 27;16:1699469. doi: 10.3389/fpls.2025.1699469 (PMC12695741; doi:10.3389/fpls.2025.1699469)
Supplement: Supplementary file 6 [file Presentation6.pptx]

## Slide 1
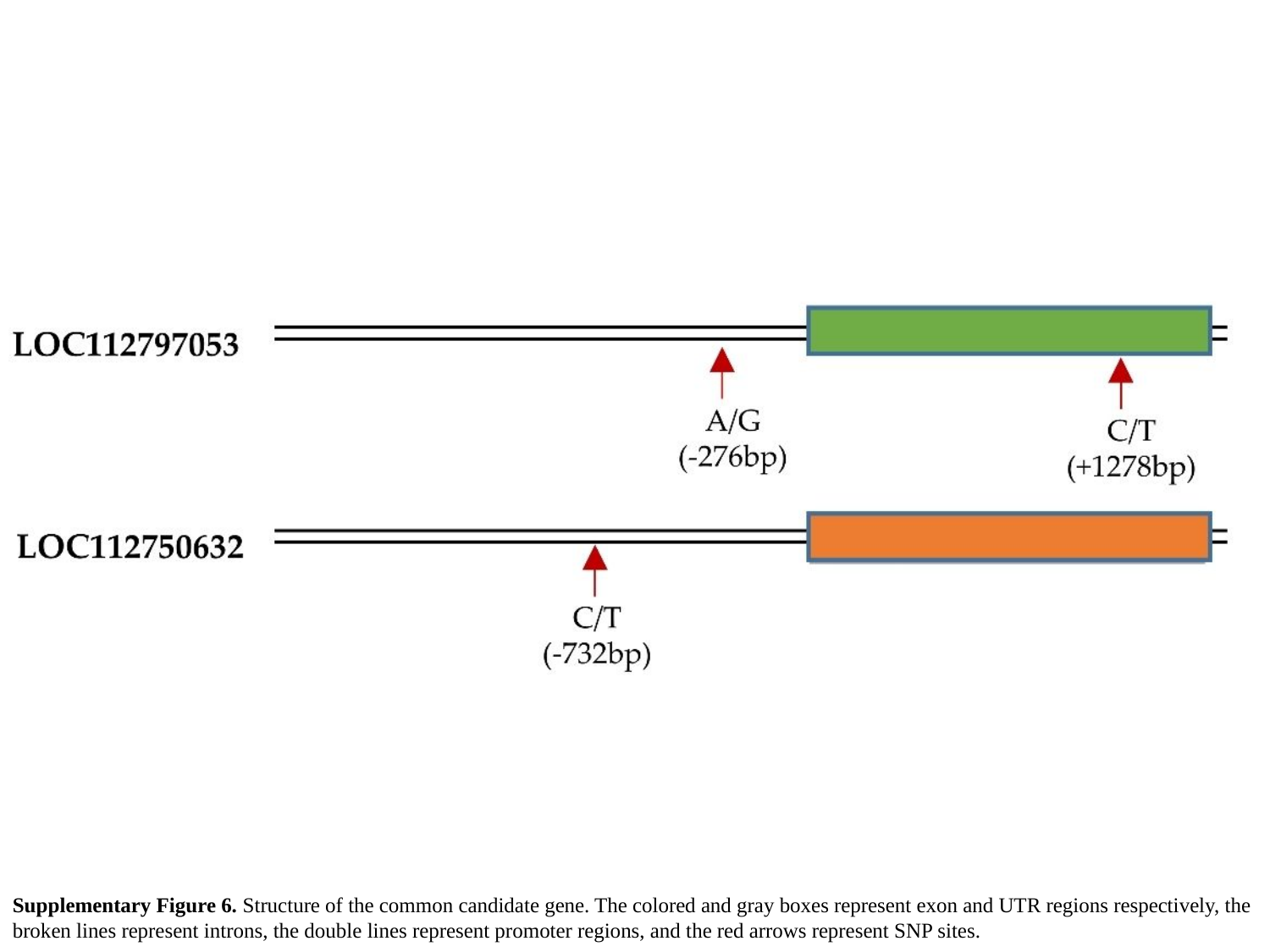

Supplementary Figure 6. Structure of the common candidate gene. The colored and gray boxes represent exon and UTR regions respectively, the broken lines represent introns, the double lines represent promoter regions, and the red arrows represent SNP sites.
