# Supplementary material for: Genome-wide association and RNA-seq analyses reveal genes linked to salt stress in peanut (Arachis hypogaea L.)
Source: Front Plant Sci. 2025 Nov 27;16:1699469. doi: 10.3389/fpls.2025.1699469 (PMC12695741; doi:10.3389/fpls.2025.1699469)
Supplement: Supplementary file 14 [file Table8.docx]

Supplementary Material

# Supplementary Data for instrument model names, company details, and URLs：

SPAD-502 Plus chlorophyll meter (Konica Minolta, Inc., Tokyo, Japan)

CMT Vibrating Sample Mill (T1-100 model, CMT Co. Ltd., Tokyo, Japan)

LAQUAtwin Na⁺ sensor (LAQUAtwin-NA-11, Horiba, Kyoto, Japan)

Epoch™ Microplate Spectrophotometer (BioTek Instruments, Winooski, VT, USA)

Sodium concentration measurement method (<https://www.horiba.com/chn/water-quality/applications/food-beverage/sodium-value-check-for-canned-food/>)

The reference genome arahy.Tifrunner.gnm1.KYV3 (https://www.ncbi.nlm.nih.gov/assembly)

R software (R Foundation for Statistical Computing, Vienna, Austria)

Origin software (https://www.originlab.com/)

Qiagen RNeasy Plant Mini Kit (Qiagen, Hilden, Germany)

NanoDrop ND-1000 spectrophotometer (Thermo Fisher Scientific Inc., Wilmington, DE, USA)

FastQC v0.12 (Babraham Institute, Cambridge, United Kingdom)

The *A. hypogaea* reference coding sequence genome (https://ftp.ncbi.nlm.nih.gov/genomes/all/GCF/003/086/295/GCF_003086295.2_arahy.Tifrunner.gnm1.KYV3/)

ClusterGVis (https://github.com/junjunlab/ClusterGVis)

SuperScript™ III First-Strand Synthesis System (Invitrogen, Carlsbad, CA, USA)

PowerUp SYBR Green Master Mix (Thermo Fisher Scientific Inc., Wilmington, DE, USA)

Applied Biosystems QuantStudio 1 Real-Time PCR System (Applied Biosystems, Foster City, CA, USA)

NCBI Primer-BLAST tool (https://www.ncbi.nlm.nih.gov/tools/primer-blast/index.cgi?LINK_LOC=BlastHome).

Excel (Microsoft, Redmond, WA, USA)

Biosearch Technologies (Teddington, Middlesex, UK)

QuantStudio™ Design & Analysis v1.5.1 (Thermo Fisher Scientific Inc., Wilmington, DE, USA)

GoTaq® G2 Green Master Mix (Promega, Madison, WI, USA)

FavorPrep™ GEL/PCR Purification Mini Kit (FAVORGEN Biotech, Princeton, NJ, USA)

ABI 3730XL Genetic Analyzer (Applied Biosystems, Foster City, CA, USA)

SnapGene v6.2.0 (<https://www.snapgene.com>)

factoextra package (<https://rpkgs.datanovia.com/factoextra/index.html>)

SPSS 15.0 (IBM Corp, Armonk, NY, USA)

pophelper package (http://pophelper.com/)
